# Higher participation rates for specific health checkups are associated with a lower incidence of treated ESKD in Japan

**DOI:** 10.1007/s10157-023-02412-3

**Published:** 2023-10-09

**Authors:** Minako Wakasugi, Ichiei Narita

**Affiliations:** 1 Department of Inter-Organ Communication Research, 1-757 Asahimachi, Chuo-ku, Niigata, 951-8510 Japan; 2 Division of Clinical Nephrology and Rheumatology, Niigata University Graduate School of Medical and Dental Sciences, Niigata, Japan

**Keywords:** Dialysis, General population, Health checkups, Regional variation, Standardized incidence ratio

## Abstract

**Background:**

A Japanese cohort study previously reported that not attending health checkups was associated with an increased risk of treated end-stage kidney disease (ESKD). The present study aimed to examine this association at the prefecture level.

**Methods:**

We conducted an ecological study of all prefectures in Japan (*n* = 47) using five sources of nationwide open data. We explored associations of participation rates for Specific Health Checkups (SHC participation rates), the estimated prevalence of chronic kidney disease (CKD), and the ratio of nephrology specialists for each prefecture with prefecture-specific standardized incidence rates (SIRs) of treated ESKD using structural equation modeling.

**Results:**

Prefecture-specific SHC participation rates ranged from 44.2% to 65.9%, and were negatively correlated with prefecture-specific SIRs and prevalence of CKD, and positively correlated with the ratio of nephrology specialists. SHC participation rates had significant negative effects on prefecture-specific SIRs (standardized estimate (*β*) = − 0.38, *p* = 0.01) and prefecture-specific prevalence of CKD (*β* = − 0.32, *p* = 0.02). Through SHC participation rates, the ratio of nephrology specialists had a significant indirect negative effect on prefecture-specific SIRs (*β*= − 0.14, *p* = 0.02). The model fitted the data well and explained 14% of the variance in SIRs.

**Conclusions:**

Our findings support the importance of increasing SHC participation rates at the population level and may encourage people to undergo health checkups.

**Supplementary Information:**

The online version contains supplementary material available at 10.1007/s10157-023-02412-3.

## Introduction

Japan, a country with one of the highest incidence and prevalence rates of treated end-stage kidney disease (ESKD) [1], has substantial regional variation in the incidence of treated ESKD [2–8] despite a uniform health care and insurance system and low ethnic and racial diversity. We previously reported associations of sex- and prefecture-specific prevalence of obesity/overweight and proteinuria with sex- and prefecture-specific standardized incidence rates (SIRs) of treated ESKD [8]. Models consisting of the prevalence of overweight/obesity, prevalence of proteinuria, and ratio of nephrology specialists explained 26% and 28% of the variance in SIRs for men and women, respectively, indicating that residual factors affect the variance in SIRs [8].

One residual factor that may affect the variance in SIRs may be participation rates for health checkups. A cohort study of Japanese adults reported that not attending health checkups was associated with an increased risk of treated ESKD, defined as the initiation of kidney replacement therapy with hemodialysis, peritoneal dialysis, or kidney transplantation [9]. Thus, prefectures with low participation rates for health checkups may have an increased incidence of treated ESKD. In fact, large variations have been observed by prefecture in participation rates for Specific Health Checkups (hereafter, SHC participation rates), an annual health screening program introduced by Japan’s Ministry of Health, Labour and Welfare since 2008 to identify individuals requiring specific health guidance for the purpose of reducing the number of people having, or at risk for, metabolic syndrome [10]. The SHC participation rate in 2008 was 38.5% and varied widely by prefecture, ranging from 27.5% to 53.3% (Supplementary Table 1).

To date, however, no study has examined the association between SHC participation rates and the incidence of treated ESKD at the prefecture level. Accordingly, this ecological study aimed to examine the relationship between prefecture-specific SHC participation rates and SIRs of treated ESKD in Japan. We also evaluated the prevalence of chronic kidney disease (CKD) in each prefecture as a potential mediator between SHC participation rates and SIRs of treated ESKD, as well as the ratio of nephrology specialists to all physicians in each prefecture as a surrogate indicator for the quality of CKD care. We tested the hypothesis that regional variation in the incidence of treated ESKD among the general Japanese population could be explained, at least in part, by prefecture-specific SHC participation rates.

## Materials and methods

### Study design and data source

This cross-sectional ecological study used data from five sources of nationwide open data: Data on SHC and Specific Health Guidance [10], the annual survey of the Japanese Society for Dialysis Therapy Renal Data Registry (JRDR), national vital statistics, National Database of Health Insurance Claims and SHC of Japan (NDB) Open Data, and Statistics of Physicians, Dentists and Pharmacists. SHC participation rates in 2019 of each prefecture were extracted from the Data on SHC and Specific Health Guidance [10] reported by the Ministry of Health, Labour and Welfare.

An incident case of treated ESKD was defined as a patient with loss of kidney function that resulted in maintenance dialysis therapy including both hemodialysis and peritoneal dialysis [11]. Given the small number of pre-emptive kidney transplant patients in Japan [12], this definition covers almost all treated ESKD patients in Japan. Incident cases of treated ESKD were extracted from annual data of the JRDR for 2020 and 2021 using the Web-based Analysis of Dialysis Data Archives (WADDA) system [13]. Details on registry data collection techniques and characteristics of this dialysis population have been described elsewhere [13, 14]. In brief, the JRDR collects data every year through questionnaire surveys sent to all dialysis facilities in Japan. The population for each prefecture during the same 2-year period was obtained from national vital statistics. SIR of treated ESKD was calculated as the ratio of the observed number of incident cases of treated ESKD patients to the expected number of incident cases of treated ESKD using the indirect method previously described [8, 15].

Numbers of people aged 40–74 years stratified by eGFR and proteinuria levels in each prefecture for 2019 were obtained from NDB Open Data [16]. Details of NDB Open Data have been described elsewhere [17]. In brief, NDB, a large national administrative claims database, refers to the National Database of Health Insurance Claims and SHC of Japan operated by the Ministry of Health, Labour and Welfare. NDB Open Data were constructed by aggregating a part of NDB without any confidential information [17]; therefore, researchers using NDB Open Data cannot access patient- or facility-level information.

The prefecture-specific prevalence of CKD was calculated as the estimated number of people aged 40–74 years with CKD divided by the total number of people aged 40–74 years in each prefecture. CKD was defined as an eGFR < 60 mL/min/1.73 m^2^ and/or proteinuria [18]. Proteinuria was defined as a dipstick urinalysis score of 1 + or greater (equivalent to ≥ 30 mg/dL). The number of people aged 40–74 years with CKD in each prefecture was estimated as follows. First, we calculated the prevalence of CKD by 5-year age bands and sex in each prefecture using data obtained from NDB Open Data [16] by dividing the number of participants with CKD by the total number of SHC participants. Next, we estimated the number of people with CKD by 5-year age bands and sex by multiplying the prevalence of CKD with the corresponding age- and sex-specific number of people in the general population in each prefecture, assuming that the prefecture-specific prevalence of CKD among participants in NDB Open Data is the same as that of the entire population. We then summed the number of people with CKD aged 40–74 years in each prefecture, and estimated the prefecture-specific prevalence of CKD by dividing the estimated number of people aged 40–74 years with CKD by the total number of people aged 40–74 years in each prefecture. Similarly, we estimated the number and prevalence of prefecture-specific CKD patients aged 60–74 years.

Numbers of nephrology specialists and all physicians in each prefecture were extracted from the Statistics of Physicians, Dentists and Pharmacists reported by the Ministry of Health, Labour and Welfare [19]. We used data from 2020 since the statistics are reported every two years. The prefecture-specific ratio of nephrology specialists to all physicians was calculated as the number of nephrology specialists divided by the total number of all physicians by prefecture.

The present study was conducted according to the principles of the Declaration of Helsinki. All data used in the analyses were anonymized and included no individual patient data. Thus, ethical review and informed consent were not required.

### Statistical analysis

Pearson’s correlation coefficients were used to evaluate relationships between variables. Hierarchical multiple regression was used to clarify further the comparative effects of prefecture-specific SHC participation rates, prefecture-specific prevalence of CKD, and prefecture-specific ratio of nephrology specialists on prefecture-specific SIRs. In Model 1, prefecture-specific SHC participation rates were first entered into the regression model as an independent variable. In Model 2, prefecture-specific prevalence of CKD was added. In Model 3, prefecture-specific ratio of nephrology specialists was added. Adjusted R-squared values were used to determine the percentage of variation explained by the independent variable(s) that affects the dependent variables. Multicollinearity of independent variables was assessed by variation inflation factor (VIF). A VIF of 10 is considered excessive, while a VIF as low as 4 indicates high levels of multicollinearity between predictor variables.

Structural equation modeling (SEM) was performed to evaluate the impact of the association between SHC participation rates, prevalence of CKD, and ratio of nephrology specialists on prefecture-specific SIRs. We developed a model for SIR that included variables based on results of the correlation analysis. Direct effects are shown as arrows originating from an independent variable leading and pointing to a dependent variable. Indirect effects are shown as a mediating variable with an arrow pointing to it from an independent variable and also an arrow pointing to another dependent variable. Indicators to evaluate goodness-of-fit of the structural equation model included the chi-square statistic, Comparative Fit Index (CFI), adjusted Goodness-of-Fit Index (AGFI), and Root Mean Square Error of Approximation (RMSEA). Our goals for model fit were: non-significant chi-square statistic, CFI > 0.90, AGFI > 0.90, and RMSEA < 0.08 [20].

All reported *P*-values were two-sided, and *P* < 0.05 was considered statistically significant. Statistical analyses were performed using AMOS version 27 (IBM Inc, Armonok, NY) and IBM SPSS Statistics version 27 (SPSS, Chicago).

## Results

The national SHC participation rate was 55.3% in 2019 (Supplementary Table 2). SHC participation rates varied widely by prefecture, ranging from 44.2% to 65.9%. Prefecture-specific SHC participation rates in 2019 were highly correlated with those in 2008 (*R*^2^ = 0.81, *p* < 0.001). Prefecture-specific SIRs also varied by prefecture, ranging from 0.73 to 1.34. In 2019, 9,420,443 adults aged 40–74 years were estimated to have CKD, which accounted for 16% of people aged 40–74 years in Japan. The estimated prevalence of CKD among people aged 40–74 years varied by prefecture, ranging from 11 to 20%. The estimated prevalence of CKD among people aged 60–74 years was 25% in Japan and also varied by prefecture, ranging from 16 to 30% (Supplementary Table 3). The prefecture-specific CKD prevalence among people aged 40–74 years was highly correlated with that among people aged 60–74 years (*R*^2^ = 0.83, *p* < 0.001). In 2020, 323,700 medical doctors worked at medical facilities in Japan, and of these, 5,360 (1.7%) were nephrology specialists. The ratio of nephrology specialists ranged from 0.2% to 2.3%.

**Fig. 1:**
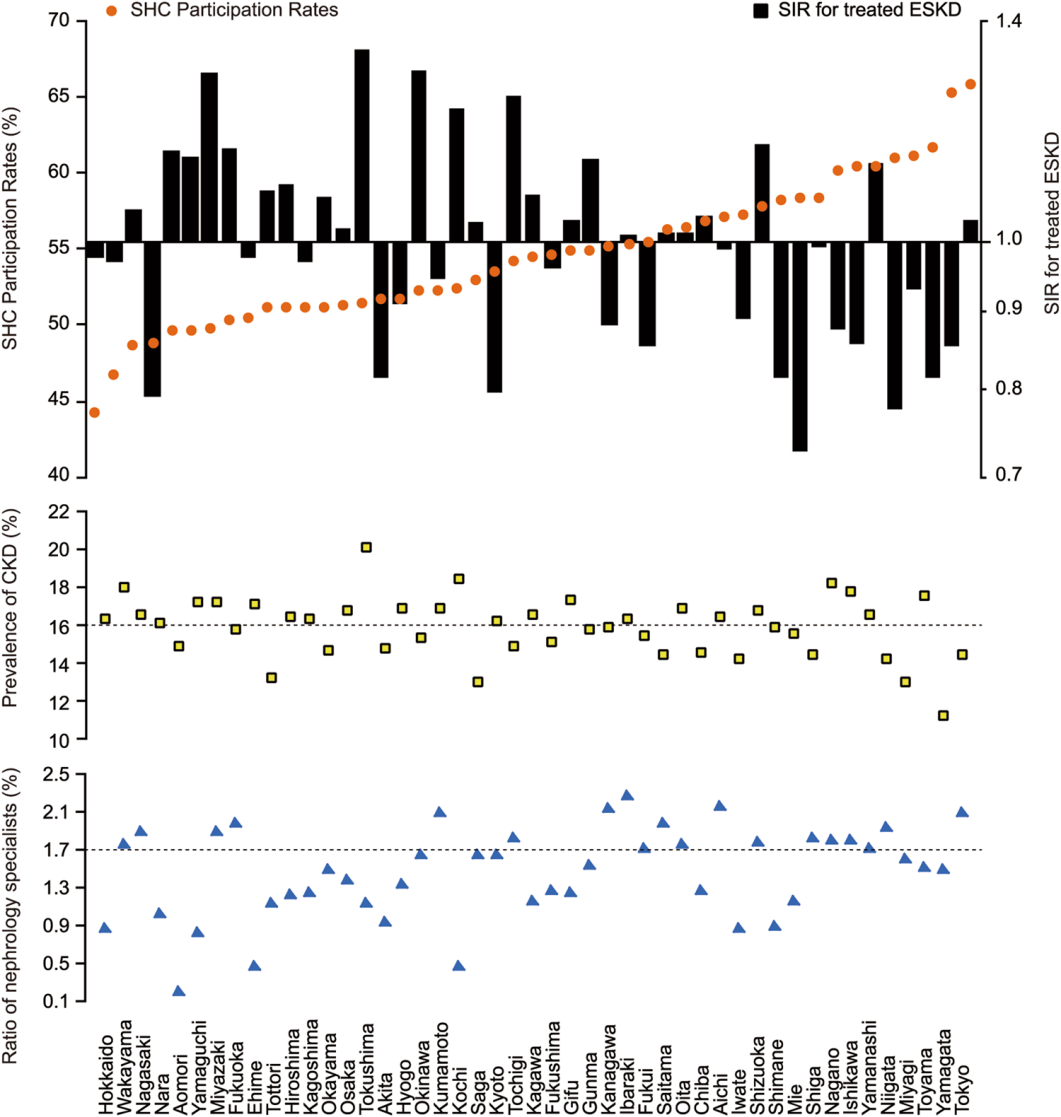
SHC participation rates, SIRs of treated ESKD, prevalence of CKD, and ratio of nephrology specialists in the 47 prefectures. Prefecture-specific SIRs are presented in ascending order. Note that the y-axis in SIR for treated ESKD is a logarithmic scale. Dashed lines show national averages. *CKD* chronic kidney disease, *ESKD* end-stage kidney disease, *SHC* specific health checkups, *SIR* standardized incidence ratio

Figure 1 shows prefecture-specific SHC participation rates in ascending order. Prefectures with high participation rates were more likely to have lower SIRs for treated ESKD than the national average (i.e., 1.0), a lower prevalence of CKD than the national prevalence (i.e., 16%), and a higher ratio of nephrology specialists than the national ratio (i.e., 1.7%).

Pearson’s correlation analysis revealed that SHC participation rates were negatively correlated with both prefecture-specific SIRs (*r* = − 0.34, *p* = 0.02) and prevalence of CKD (*r* = − 0.32, *p* = 0.03), but positively correlated with the ratio of nephrology specialists (*r* = 0.36, *p* = 0.01) (Table 1). No significant correlations were observed between prefecture-specific SIRs, prevalence of CKD, and ratios of nephrology specialists (Supplementary Figure). Furthermore, no significant association was observed between the prefecture-specific prevalence of CKD among people aged 60–74 years and prefecture-specific SIRs (*r* = 0.24, *p* = 0.11).

**Table 1:** Pearson correlation analysis between variables

Variables	SHC participation rates	SIR for treated ESKD	Prevalence of CKD	Ratio of nephrology specialists
SHC participation rates	1			
SIR for treated ESKD	− 0.34 0.02	1		
Prevalence of CKD	− 0.32 0.03	0.23 0.12	1	
Ratio of nephrology specialists	0.36 0.01	− 0.03 0.82	− 0.07 0.64	1

The upper value is the linear correlation coefficient and the lower value is the *p*-value

*CKD* chronic kidney disease, *ESKD* end-stage kidney disease, *SHC* Specific Health Checkups, *SIR* standardized incidence ratio

Hierarchical linear regression analysis revealed that SHC participation rates were significantly and negatively associated with prefecture-specific SIRs (Table 2). Further adjusting for the prevalence of CKD (Model 2) and ratio of nephrology specialists (Model 3) did not change the association between SHC participation rates and prefecture-specific SIRs.

**Table 2:** Hierarchical regression analysis for variables predicting SIRs

Variables	Model 1		Model 2		Model 3	
	*β*	*P*	*β*	*P*	*β*	*P*
SHC participation rates	− 0.34	0.02	− 0.30	0.05	− 0.33	0.04
Prevalence of CKD			0.14	0.36	0.13	0.38
Ratio of nephrology specialists					0.10	0.53
Adjusted *R*^2^	0.10		0.09		0.08	
F for change in *R*^2^	5.94	0.02	0.85	0.36	0.40	0.53

N for all variables = 47

*β*standardized regression coefficient, *CKD* chronic kidney disease, *SHC* specific health checkups, *SIR* standardized incidence ratio

**Fig. 2:**
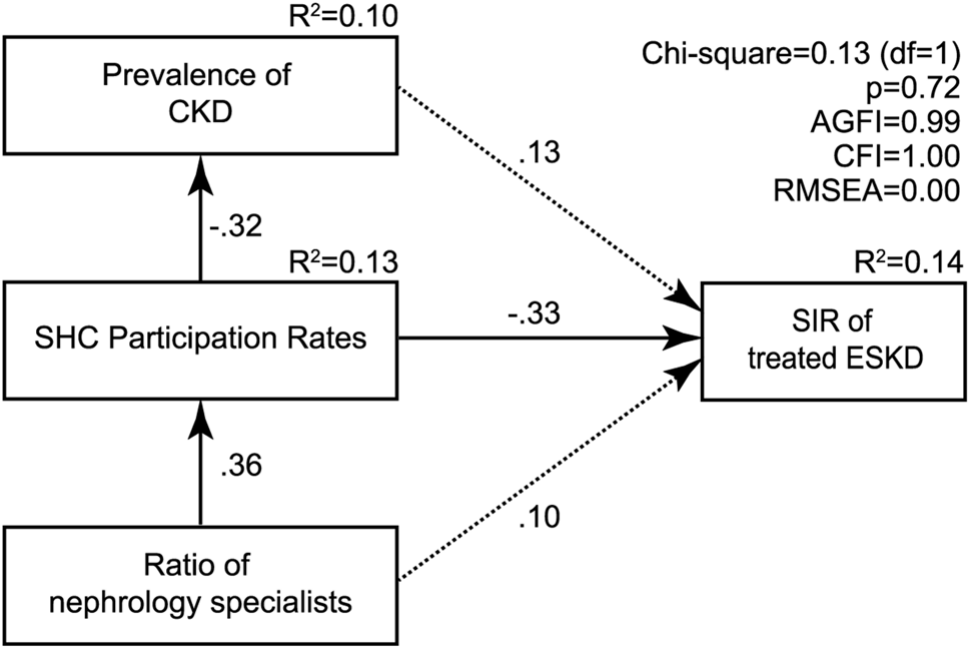
SEM models and standardized estimates. Solid lines indicate significant paths. Dashed lines indicate non-significant paths. The standardized regression coefficient is provided for each relationship. For simplicity, residual variance associated with independent variables is not represented. *CKD* chronic kidney disease, *ESKD* end-stage kidney disease, *SEM* structural equation modelling, *SIR* standardized incidence ratio

Based on these results, a hypothetical model was constructed (Fig. 2). SHC participation rates had a significant total effect on prefecture-specific SIRs (*β*= − 0.38, *p* = 0.01), which was the sum of a direct negative effect on prefecture-specific SIRs (*β*= − 0.33, *p* = 0.03) and an indirect negative effect through the prefecture-specific prevalence of CKD (*β* = − 0.04, *p* = 0.33) (Table 3). SHC participation rates also had a significant direct negative effect on the prefecture-specific prevalence of CKD (*β* = − 0.32, *p* = 0.02). The direct path from the prefecture-specific prevalence of CKD to prefecture-specific SIRs, however, was not significant (*β *= 0.13, *p* = 0.36). The ratio of nephrology specialists had a significant indirect negative effect through SHC participation rates (*β* = − 0.14, *p* = 0.02) and a non-significant direct positive effect on prefecture-specific SIRs (*β*= 0.10, *p* = 0.51), resulting in a non-significant total effect on prefecture-specific SIRs (*β* = − 0.04, *p* = 0.71). The fit of the model to the data was excellent (chi-square value = 0.13, df = 1, *p* = 0.72, CFI = 1.00, AGFI = 0.99, and RMSEA = 0.00), and this model explained 14% of the variance in prefecture-specific SIRs. When using the prefecture-specific prevalence of CKD among people aged 60–74 years instead of that among people aged 40–74 years, the fit of the SEM model to the data was poor (chi-square value = 2.129, df = 1, *p* = 0.14, CFI = 0.91, AGFI = 0.78, RMSEA = 0.16).

**Table 3:** Structural equation modeling: standardized direct, indirect, and total effects

Paths	β	*P*
SHC participation rates → SIR of treated ESKD		
Standardized direct effect	− 0.33	0.03
Standardized indirect effect	− 0.04	0.33
Standardized total effect	− 0.38	0.01
Ratio of nephrology specialists → SIR of treated ESKD		
Standardized direct effect	0.10	0.51
Standardized indirect effect	− 0.14	0.02
Standardized total effect	− 0.04	0.71

N for all variables = 47

*β*standardized regression coefficient, *ESKD* end-stage kidney disease, *SHC* specific health checkups, *SIR* standardized incidence ratio

## Discussion

This ecological study using SEM revealed that SHC participation rates had significant direct negative effects on prefecture-specific SIRs and prefecture-specific prevalence of CKD. Furthermore, through SHC participation rates, the ratio of nephrology specialists had a significant indirect negative effect on prefecture-specific SIRs, suggesting that a higher prefecture-specific ratio of nephrology specialists was associated with lower prefecture-specific SIRs. The SEM model explained 14% of the variance in prefecture-specific SIRs, indicating that prefecture-specific SHC participation rates can partially explain regional variation in prefecture-specific SIRs of treated ESKD.

SHC participation rates had a significant impact on prefecture-specific SIRs and prevalence of CKD, suggesting that increasing SHC participation rates could potentially have a preventive effect on CKD and ESKD in the general population. This is in line with the Neyagawa Health checkups and Health care in Kokuho database (NHHK) study, which showed that men who did not attend health checkups and did not undergo a kidney test using dipstick urinalysis and/or serum creatinine measurement at medical facilities were at significantly higher risk of treated ESKD than those who attended checkups, especially among those aged ≥ 75 years [9]. Although SHCs focus on metabolic syndrome rather than CKD, preventing metabolic syndrome could reduce the likelihood of developing CKD and ESKD. A recent review of randomized controlled trials and observational studies reported that general health checks were associated with increased chronic disease recognition and treatment, risk factor control, preventive service uptake, and improved patient-reported outcomes [21]. The review reported that general health checks were sometimes associated with modest improvements in health behaviors, such as physical activity and diet [21]. Health behaviors are significantly associated with incident diabetes, hypertension, and CKD in the general population [22–24]. These reports and our present findings, taken together, suggest the possibility that increasing SHC participation rates can reduce the incidence of treated ESKD.

Although SHC participation rates had a significant direct negative effect on the prefecture-specific prevalence of CKD, the path from prevalence of CKD to prefecture-specific SIRs was not significant. This may be because the prevalence of CKD was estimated among people aged 40–74 years who participated in SHCs, which may not be the same as that of the general population in each prefecture. Although we assumed that the prefecture-specific prevalence of CKD among participants in NDB Open Data is the same as that of the entire population, this may not be the case. According to the NHHK study, men who did not attend health checkups were at significantly higher risk of treated ESKD than those who attended checkups [9], suggesting that the prevalence of CKD among SHC participants would be lower than that among non-participants. If this is true, our calculations may have underestimated the prevalence of CKD and this may explain why the path from the prevalence of CKD to prefecture-specific SIRs was not significant.

We found that the ratio of nephrology specialists had a significant indirect path to prefecture-specific SIRs through SHC participation rates, indicating that a higher prefecture-specific ratio of nephrology specialists was associated with lower prefecture-specific SIRs via increased SHC participation rates. This could be explained by measures against CKD taken by the Japanese government starting in 2008 and recently updated in 2018 [25]. Since one of the aims of the measures is to prevent CKD exacerbation through early detection and diagnosis of CKD, prefectures with a high ratio of nephrology specialists may be more active in encouraging people to undergo health checkups to prevent both CKD and ESKD.

The national SHC participation rate increased from 38.5% in 2008 to 55.3% in 2019, although large variations were observed by prefecture in 2019, ranging from 44.2% to 65.9%. Prefecture-specific SHC participation rates in 2019 were highly correlated with those in 2008, suggesting that maintaining high SHC participation rates for years may be associated with a decreased incidence of treated ESKD and a decreased prevalence of CKD.

This study has several strengths. First, since data were extracted from nationwide sources, our findings are broadly generalizable to the Japanese population. Second, to the best of our knowledge, this is the first study to report associations of prefecture-specific SHC participation rates with prefecture-specific SIRs of treated ESKD. Our findings provide evidence that low SHC participation rates are associated with a high incidence of treated ESKD from a population-level perspective.

There are also several limitations worth noting. First, although we targeted all 47 prefectures in Japan (*n* = 47), we could only use a limited number of parameters to estimate SEM models. Second, only patients who had undergone dialysis treatment were included, as data were not available for patients who had not initiated dialysis or had undergone pre-emptive kidney transplantation in Japan. The number of pre-emptive kidney transplants in Japan, however, is small, with only 662 patients being reported in 2017 [12]. In addition, data were unavailable for patients with ESKD who did not receive renal replacement therapy. Third, we assumed that the prefecture-specific prevalence of CKD among participants in NDB Open Data is the same as that of the entire population. However, no data were available about the prevalence of CKD among non-participants. Finally, since this was an ecological study, there is a risk of ecological fallacy, i.e., findings observed at the prefecture level may not be seen at the individual level. However, the NHHK study showed that not attending health checkups was associated with an increased risk of ESKD even at the individual level [9], suggesting that increasing SHC participation rates is important for preventing ESKD at both the individual and prefecture levels.

## Conclusions

This ecological study showed that higher prefecture-specific SHC participation rates are associated with lower prefecture-specific SIRs of treated ESKD. Our findings provide evidence to support the importance of increasing SHC participation rates from a population-level perspective and may encourage people to undergo health checkups.

### Supplementary Information

Below is the link to the electronic supplementary material.

Supplementary file1 (PDF 636 KB)
