# Supplementary material for: Higher participation rates for specific health checkups are associated with a lower incidence of treated ESKD in Japan
Source: Clin Exp Nephrol. 2023 Oct 9;28(3):201–7. doi: 10.1007/s10157-023-02412-3 (PMC10881630; doi:10.1007/s10157-023-02412-3)
Supplement: Supplementary file 1 — Supplementary file1 (PDF 636 KB) [file 10157_2023_2412_MOESM1_ESM.pdf]

Supplementary Table 1. SHC participation rates in 2008 by prefecture

Prefecture	SHC participation rates in 2008 [a]
Hokkaido	29.2%
Aomori	33.9%
Iwate	37.4%
Miyagi	48.1%
Akita	35.4%
Yamagata	44.9%
Fukushima	40.3%
Ibaraki	36.8%
Tochigi	33.6%
Gunma	40.8%
Saitama	37.9%
Chiba	39.1%
Tokyo	53.3%
Kanagawa	37.0%
Niigata	45.5%
Toyama	46.7%
Ishikawa	38.5%
Fukui	35.4%
Yamanashi	40.9%
Nagano	42.4%
Gifu	39.2%
Shizuoka	38.6%
Aichi	40.3%
Mie	38.5%
Shiga	39.5%
Kyoto	38.1%
Osaka	34.2%
Hyogo	35.4%
Nara	30.4%
Wakayama	27.5%
Tottori	33.5%
Shimane	41.8%
Okayama	35.0%

Hiroshima	33.3%
Yamaguchi	32.4%
Tokushima	36.1%
Kagawa	41.4%
Ehime	32.9%
Kochi	34.0%
Fukuoka	34.5%
Saga	35.1%
Nagasaki	32.9%
Kumamoto	36.5%
Oita	41.4%
Miyazaki	33.0%
Kagoshima	29.1%
Okinawa	34.5%
All Japan	38.5%

SHC, Specific Health Checkup.

[a] Reproduced from Data on Specific Health Checkups and Specific Health Guidance reported by the Ministry of Health, Labour and Welfare [10].

Supplementary Table 2. SHC participation rates, SIR of treated ESKD, prevalence of CKD, and ratio of nephrology specialists in each prefecture

Prefecture	SHC participation rates [a]	SIR of treated ESKD	Estimated number of CKD patients	Estimated prevalence of CKD	Number of nephrology specialists [b]	Ratio of nephrology specialists
Hokkaido	44.2%	0.98	423758	16%	115	0.9%
Aomori	49.6%	1.15	93307	15%	6	0.2%
Iwate	57.3%	0.89	85230	14%	22	0.9%
Miyagi	61.2%	0.93	142249	13%	92	1.6%
Akita	51.7%	0.82	72288	15%	22	0.9%
Yamagata	65.2%	0.86	58611	11%	37	1.5%
Fukushima	54.7%	0.96	136466	15%	48	1.3%
Ibaraki	55.4%	1.01	227774	16%	127	2.3%
Tochigi	54.2%	1.25	140901	15%	84	1.8%
Gunma	54.9%	1.13	147371	16%	70	1.5%
Saitama	56.3%	1.02	509166	15%	261	2.0%
Chiba	56.9%	1.04	440054	15%	166	1.3%
Tokyo	65.9%	1.03	922106	15%	951	2.1%
Kanagawa	55.1%	0.88	692205	16%	442	2.1%
Niigata	61.1%	0.78	153175	14%	88	2.0%
Toyama	61.7%	0.81	89088	18%	41	1.5%
Ishikawa	60.4%	0.86	96109	18%	60	1.8%
Fukui	55.5%	0.86	55621	15%	34	1.7%
Yamanashi	60.5%	1.13	65114	17%	35	1.7%
Nagano	60.2%	0.88	177717	18%	91	1.8%
Gifu	54.9%	1.03	164585	17%	56	1.3%
Shizuoka	57.8%	1.16	293525	17%	142	1.8%
Aichi	57.2%	0.99	569667	16%	367	2.2%
Mie	58.3%	0.73	131874	16%	48	1.2%
Shiga	58.4%	0.99	94435	15%	61	1.8%
Kyoto	53.5%	0.80	193747	16%	142	1.7%
Osaka	51.3%	1.02	692930	17%	354	1.4%
Hyogo	51.7%	0.91	441737	17%	197	1.4%
Nara	48.8%	0.79	102869	16%	38	1.0%
Wakayama	46.8%	0.97	80771	18%	50	1.8%
Tottori	51.1%	1.08	34981	13%	20	1.1%
Shimane	58.2%	0.82	50263	16%	18	0.9%
Okayama	51.2%	1.07	127468	15%	91	1.5%
Hiroshima	51.2%	1.09	216802	16%	93	1.2%
Yamaguchi	49.6%	1.14	111502	17%	29	0.8%

Tokushima	51.5%	1.34	71666	20%	28	1.1%
Kagawa	54.5%	1.07	75800	17%	32	1.2%
Ehime	50.4%	0.98	110927	17%	18	0.5%
Kochi	52.5%	1.22	62192	18%	11	0.5%
Fukuoka	50.3%	1.15	373508	16%	316	2.0%
Saga	53.0%	1.03	49125	13%	39	1.7%
Nagasaki	48.7%	1.05	105910	17%	80	1.9%
Kumamoto	52.3%	0.95	136486	17%	109	2.1%
Oita	56.4%	1.01	90427	17%	57	1.8%
Miyazaki	49.8%	1.29	87791	17%	52	1.9%
Kagoshima	51.2%	0.97	122763	16%	57	1.3%
Okinawa	52.2%	1.30	98380	15%	63	1.7%
All Japan	55.3%	1.00	9420443	16%	5360	1.7%

CKD, chronic kidney disease; ESKD, end-stage kidney disease; SHC, Specific Health Checkup; SIR, standardized incidence ratio.

[a] Reproduced from Data on Specific Health Checkups and Specific Health Guidance reported by the Ministry of Health, Labour and Welfare [10].

[b] Reproduced from Statistics of Physicians, Dentists and Pharmacists reported by the Ministry of Health, Labour and Welfare [19].

Supplementary Table 3. Estimated number and prevalence of CKD patients aged 60-74 years in 2019 by prefecture

Prefecture	Estimated number of CKD patients aged 60-74 years	Estimated prevalence of CKD patients aged 60-74 years
Hokkaido	280553	24%
Aomori	62139	21%
Iwate	55125	20%
Miyagi	94958	20%
Akita	48702	20%
Yamagata	39187	16%
Fukushima	93834	22%
Ibaraki	155798	25%
Tochigi	96506	23%
Gunma	95146	23%
Saitama	328706	23%
Chiba	281893	23%
Tokyo	545059	25%
Kanagawa	413754	26%
Niigata	99033	20%
Toyama	58423	26%
Ishikawa	61845	27%
Fukui	36749	23%
Yamanashi	42625	25%
Nagano	117539	28%
Gifu	109661	27%
Shizuoka	195422	26%
Aichi	350346	26%
Mie	90571	25%
Shiga	61217	23%
Kyoto	124987	25%
Osaka	442766	27%
Hyogo	292175	27%
Nara	69304	24%
Wakayama	55790	27%
Tottori	24437	20%

Shimane	33797	22%
Okayama	85844	22%
Hiroshima	150207	27%
Yamaguchi	78196	26%
Tokushima	49914	30%
Kagawa	52711	25%
Ehime	77625	26%
Kochi	42657	26%
Fukuoka	251960	25%
Saga	32198	18%
Nagasaki	73970	24%
Kumamoto	93912	25%
Oita	63621	25%
Miyazaki	60688	25%
Kagoshima	84320	23%
Okinawa	61204	24%
All Japan	6117076	25%

CKD, chronic kidney disease.

Supplementary Figure

Scatter plots and histograms between SHC Participation Rates, SIR of treated ESKD, prevalence of CKD, and Ratio of nephrology specialists in the 47 prefectures.

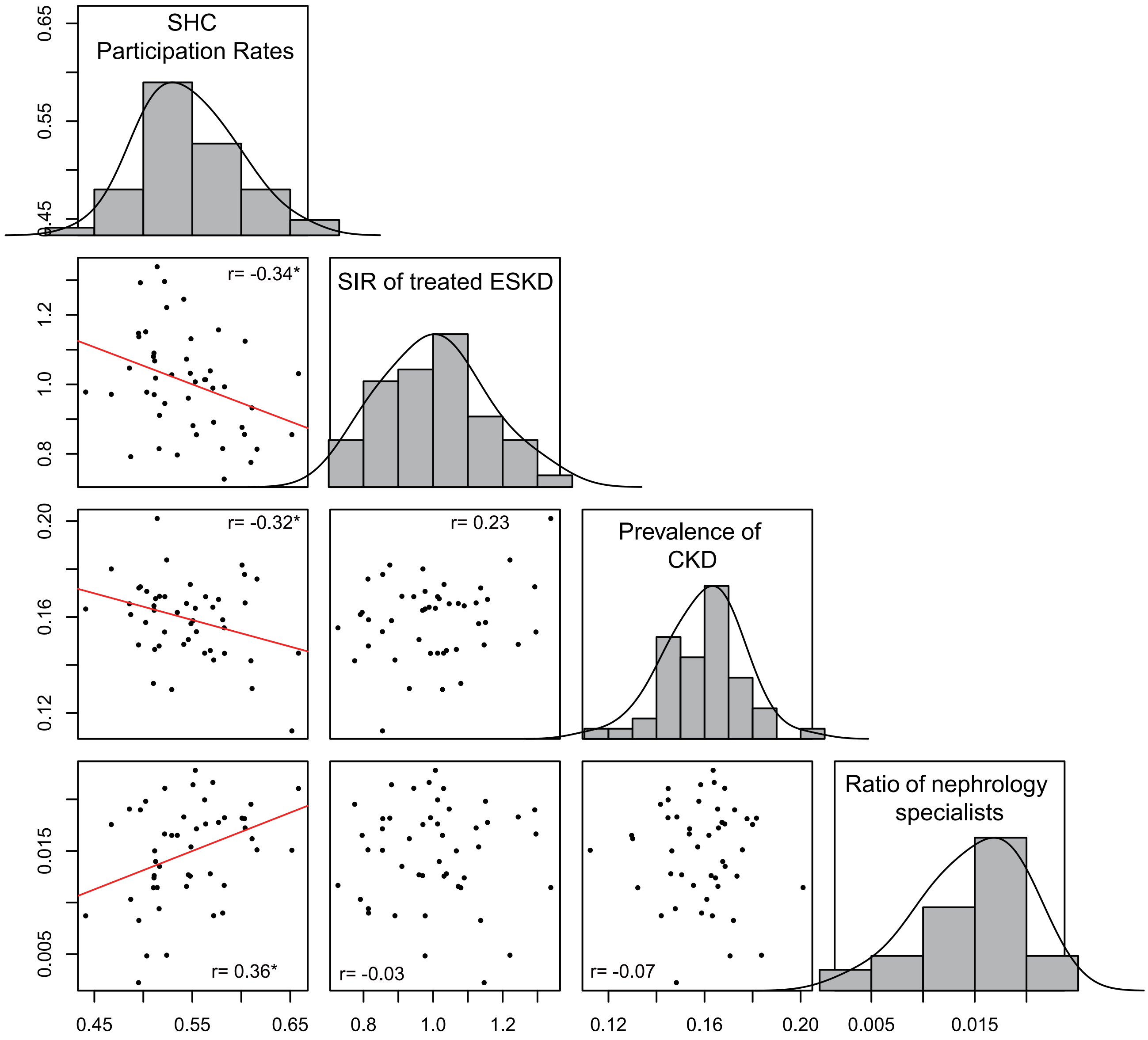

*Denotes significant Pearson’s correlation coefficients with P < 0.05.
CKD, chronic kidney disease; ESKD, end-stage kidney disease, SHC, Specific Health CHeckups; SIR standardized incidence ratio.
